# Iron deposition in gastric black spots: Clinicopathological insights and NanoSuit‐correlative light and electron microscopy analysis

**DOI:** 10.1002/deo2.398

**Published:** 2024-06-17

**Authors:** Kiichi Sugiura, Hideya Kawasaki, Takatoshi Egami, Masanao Kaneko, Natsuki Ishida, Satoshi Tamura, Shinya Tani, Mihoko Yamade, Yasushi Hamaya, Satoshi Osawa, Hiroya Takeuchi, Satoshi Baba, Ken Sugimoto, Moriya Iwaizumi

**Affiliations:** ^1^ First Department of Medicine Hamamatsu University School of Medicine Shizuoka Japan; ^2^ Institute for NanoSuit Research Preeminent Medical Photonics Education and Research Center Hamamatsu University School of Medicine Shizuoka Japan; ^3^ Department of Endoscopic and Photodynamic Medicine Hamamatsu University School of Medicine Shizuoka Japan; ^4^ Department of Surgery Hamamatsu University School of Medicine Shizuoka Japan; ^5^ Department of Diagnostic Pathology Hamamatsu University School of Medicine Shizuoka Japan; ^6^ Department of Laboratory Medicine Hamamatsu University School of Medicine Shizuoka Japan

**Keywords:** deposition, gastric, gastrointestinal endoscopy, *Helicobacter pylori*, iron

## Abstract

**Objectives:**

Black spots (BSs) are lentiginous findings observed in the gastric body and fundus during upper gastrointestinal endoscopy and are predominantly seen in patients undergoing *Helicobacter pylori* eradication treatment. However, the detailed patient background and exact composition are poorly understood. This study aims to clarify the clinicopathological features of BSs, examine patient demographics, and use the NanoSuit‐correlative light and electron microscopy (CLEM) method combined with scanning electron microscopy‐energy dispersive X‐ray spectroscopy for elemental analysis.

**Methods:**

Patients who underwent upper gastrointestinal endoscopy between 2017 and 2022 were included. Data on age, medications, blood tests, and *H. pylori* infection status were retrospectively gathered from medical records. Univariate analysis was conducted to examine BS presence, with results then used in a multivariate model to identify associated risk factors. Additionally, pathological specimens from patients with BSs were analyzed for elemental composition using the NanoSuit‐CLEM method combined with scanning electronmicroscopy‐energy dispersive X‐ray spectroscopy.

**Results:**

An analysis of 6778 cases identified risk factors for BSs, including older age and using proton pump inhibitors, statins, corticosteroids, and antithrombotic drugs. Endoscopically, BSs correlated with higher gastric atrophy and lower active *H. pylori* infection. Iron deposition at BS sites was specifically identified using NanoSuit‐CLEM.

**Conclusions:**

BSs on gastrointestinal endoscopy may indicate an absence of active *H. pylori* inflammation. The discovery of iron deposition within BSs using the NanoSuit‐CLEM method has offered new insights into the possible causative factors and advances our understanding of the etiology of BSs, bringing us closer to unraveling the underlying mechanisms of their formation.

## INTRODUCTION

Black spots (BSs) manifest as lentiginous anomalies identifiable in the gastric body and fundus during gastroscopic examination.^1^ Histological examination via light microscopy reveals the presence of brownish deposits within the lumina of fundic gland cysts. Notably, BSs are detected in 6.2% of individuals subjected to gastroscopy, with a pronounced prevalence among those receiving *Helicobacter pylori* eradication therapy or proton pump inhibitor (PPI) medication.^2^, ^3^ Moreover, BSs are commonly identified adjacent to gastric adenocarcinomas of the fundic gland type, thereby providing a significant adjunct in the diagnostic process of these specific gastric neoplasms.^4^, ^5^, ^6^

Despite the scrutiny of BSs through immunohistochemical staining and polarized light microscopy, their molecular composition and structure remain unknown.^1^ Various methodologies, including mass spectrometry, proteomic profiling, and electron spin resonance, have characterized deposits in pathological samples. However, the definitive identification of these compounds frequently proves challenging. Against this background, we have developed certain substances that polymerize when exposed to electron or plasma irradiation on the epidermis of specific insects, forming a nanometer‐thick protective layer known as the “NanoSuit.” This innovation has demonstrated the feasibility of preserving diminutive organisms during scanning electron microscopy (SEM) analysis.^7^ We have harnessed this technique to pioneer a novel investigative approach called the NanoSuit‐correlative light and electron microscopy (NanoSuit‐CLEM) and have further integrated energy dispersive X‐ray spectroscopy (EDS) analysis into this approach.^8^, ^9^ Utilizing this technique, we have successfully identified lanthanum phosphate deposits within gastric tissue.^10^ Nevertheless, there are no studies in the literature that have used this innovative technique for the analysis of BSs.

This study's primary objective is to understand the clinicopathological characteristics of BSs comprehensively. This involves a detailed analysis of patient backgrounds, including the status of *H. pylori* infection and detailed pharmacological histories. Additionally, the study aims to employ the NanoSuit‐CLEM method combined with SEM‐EDS for elemental analysis. This approach is expected to provide a deeper insight into the composition and potential causative factors of BSs, thereby contributing significantly to the understanding of these clinical findings.

## METHODS

### Participants

This retrospective study included all 8,358 adult individuals who underwent upper gastrointestinal endoscopy for abdominal symptoms or gastric cancer monitoring at Hamamatsu University Hospital between January 2017 and May 2022. Exclusions were made for cases involving postoperative stomach evaluations and instances of poor observation quality. The study was approved by the Institutional Review Board of the Hamamatsu University School of Medicine (approval no. 22‐014, 23–239), which confirmed that the study complied with the ethical guidelines of the Helsinki Declaration.

### Demographic and clinical profiles

We collected data related to age, gender, pharmaceutical regimen, blood test results within 3 months prior to endoscopy, *H. pylori* infection status, and the detection of BSs in the gastric region during endoscopy from patient medical records. Medications were categorized as those administered consistently for a minimum of 1 month, extending back 2 years from the date of endoscopy. The medication categories included H2 blockers, PPIs (vonoprazan and other PPIs), statins, corticosteroids, antiplatelet agents, anticoagulants, and iron preparations (drug details are summarized in Table S1). Regarding hematological evaluations, data for serum creatinine, hemoglobin, serum iron, serum ferritin, total cholesterol, and HbA1c were collected from the patient's medical records.

### Endoscopic assessment

For patients who met the inclusion criteria, all recorded endoscopic images were reviewed for the detection of BSs. The presence or absence of BSs was collaboratively determined by three experienced endoscopists through discussion. Cases in which differentiation between hematin and BS was difficult in the lentigo findings were excluded. Patients with ≥10 spots were classified into the diffuse group and those with <10 spots were defined as the non‐diffuse group. We evaluated the degree of gastric mucosal atrophy using the Kimura–Takemoto classification and categorized it as follows: C‐0 and C‐1 for no atrophy; C‐2 and C‐3 for closed atrophy; O‐1, O‐2, and O‐3 for opened atrophy.^11^, ^12^

### *H. pylori* Infection Status

The determination of *H. pylori* infection status was based on a combination of rapid urease test, urea breath test, stool antigen test, and biopsy culture results, alongside the patient's history of eradication treatment. According to previous research, cases exhibiting atrophic gastric mucosa alongside positive test results were classified as current *H. pylori* infections.^13^, ^14^, ^15^ In contrast, those with a history of eradication therapy or those exhibiting mucosal atrophy alongside negative test outcomes were categorized as post‐eradication cases. Cases without a history of eradication therapy and with a regular arrangement of collecting venules observed in the gastric angle were counted as uninfected.

### NanoSuit‐CLEM Method Combined with SEM‐EDS

We performed histopathological analysis using hematoxylin and eosin staining on biopsy and surgical specimens of cases where BSs were observed. For specimens with brownish substance deposits detected by the hematoxylin and eosin staining and suspected of BS, the NanoSuit‐CLEM method combined with SEM‐EDS was further used. The procedure involved deparaffinizing the sections with xylene and rehydration with a surface shield enhancer (SSE) solution.^8^, ^9^ The sections were then centrifuged at 2000 rpm for 15 s, facilitating the spin‐coating of the sections and the removal of surplus SSE solution. Post‐centrifugation, the samples were immediately introduced into the SEM and exposed to an electron beam to induce the formation of NanoSuit. Elemental composition was analyzed employing an SEM (TM4000Plus; HITACHI; accelerating voltage: 15 kV) outfitted with EDS (X‐stream‐2; Oxford Instruments). The EDS analysis was conducted using AZtecOne software (Oxford Instruments).

EDS analysis was performed on glandular ducts with BS, dilated glandular ducts without BS, and normal mucosa. The weight concentration of each detected element was quantified and articulated as a weight percent (wt%), delineating the relative concentration of the element within the analyzed area. Comparative analysis of wt% across different regions was executed.

### Statistical evaluation

All statistical evaluations were conducted using EZR (Saitama Medical Center, Jichi Medical University).^16^ The Mann–Whitney U and Fisher's exact probability tests were used to compare patient backgrounds between those with and without BSs. Variables identified as significant in the univariate analysis were incorporated into a multivariate model, and independent risk factors associated with the presence of BSs were determined through logistic regression analysis.

The wt% data derived from the EDS analysis were statistically scrutinized utilizing the Mann–Whitney U test. We determined that the results were statistically significant if the *p*‐value was below 0.05.

## RESULTS

### Patient background

**FIGURE 1:**
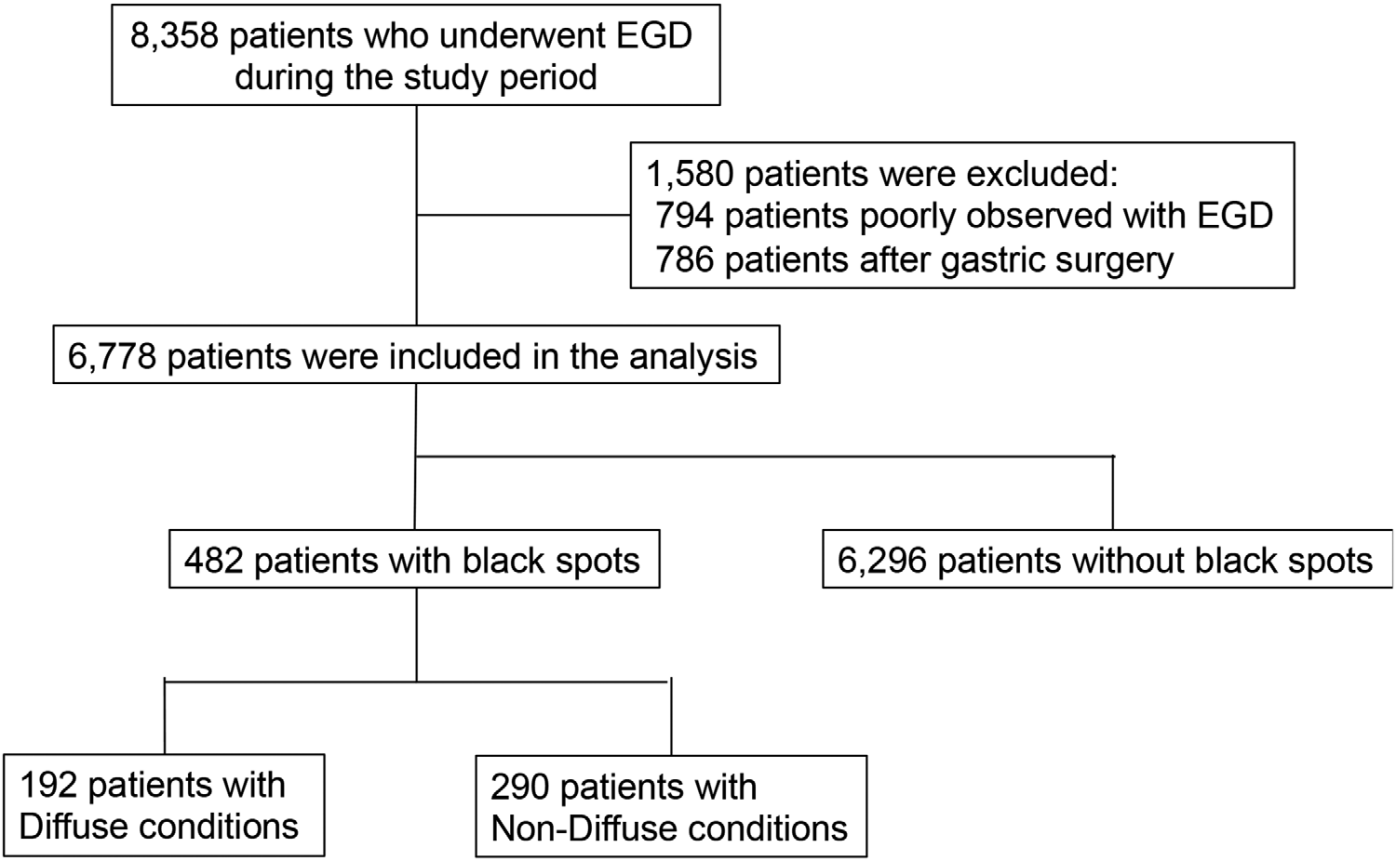
Flow chart of patients recruited in this study. EGD, esophagogastroduodenoscopy.

During the study period, 8358 patients underwent upper gastrointestinal endoscopy. Of these, 6778 were considered eligible for analysis after excluding 794 patients with poor observation and 786 patients after gastrectomy (Figure 1). The demographics of these patients are detailed in Table 1. The cohort comprised 42.3% female patients, with an average age of 69 years (interquartile range [IQR], 57–76 years). Among them, 482 patients (7.1%) had BSs, with 192 (39.8%) categorized into the diffuse group and 290 (60.2%) into the non‐diffuse group. During the examination of BS locations, 82.0% (395 out of 482) were located in normal mucosal sites, 10.4% (50 out of 482) were situated within fundic gland polyps, and 7.7% (37 out of 482) were found in both normal mucosa and fundic gland polyps.

**TABLE 1:** Characteristics of patients.

	Overall, *n* = 6778
Sex, *n* (%)	
Male	3912 (57.7)
Female	2866 (42.3)
Age, years (median [IQR])	69.0 (57.0–76.0)
H2 blocker use, *n* (%)	381 (5.6)
PPI use, *n* (%)	2084 (30.7)
Vonoprazan, *n* (%)	303 (4.5)
PPIs (excluding vonoprazan), *n* (%)	1050 (15.5)
Both drugs, *n* (%)	731 (10.8)
Statin use, *n* (%)	949 (14.0)
Corticosteroid use, *n* (%)	373 (5.5)
Antiplatelet drug use, *n* (%)	143 (2.1)
Anticoagulant drug use, *n* (%)	359 (5.3)
Iron preparation use, *n* (%)	290 (4.3)
Atrophic border, *n* (%)	
None	3126 (46.4)
Closed‐type	1523 (22.6)
Open‐type	2086 (31.0)
Hp infection status, *n* (%)	
None	3063 (45.2)
Past infection	1455 (21.5)
Current infection	623 (9.2)
Unknown	1637 (24.2)
Cr, mg/dL (median [IQR])^†^	0.8 (0.7–1.0)
Hb, g/dL (median [IQR])^†^	12.9 (11.2–14.2)
Serum iron, µg/dL (median [IQR])^†^	70.0 (43.00–100.0)
Serum ferritin, ng/mL (median [IQR])^†^	48.0 (20.0–126.8)
Chol, mg/dL (median [IQR])^†^	189.0 (164.0–216.0)
HbA1c, % (median [IQR])^†^	5.9 (5.6–6.5)

Abbreviations: Chol, cholesterol; Cr, creatinine; Hb, hemoglobin; HbA1c, hemoglobin A1c; Hp, *Helicobacter pylori*; IQR, interquartile range; PPI, proton pump inhibitor.

^†^ The test values displayed are based on measurements from the following number of patients: Cr from 5789, Hb from 5830, serum iron from 1085, serum ferritin from 650, chol from 2601, and HbA1c from 2220 patients. Patients whose data is not shown did not undergo the testing, and the data were not collected.

### Univariate analysis

As shown in Table 2, several findings were observed in the univariate analysis comparing patients with gastric BSs and those without gastric BSs. Individuals with BSs were generally older, with a median age of 75.0 years (IQR: 68.0–80.0) as opposed to 69.0 years (IQR: 55.0–76.0) for those without BSs (*p* < 0.001). Regarding medication, the usage of PPIs, statins, and corticosteroids was notably higher in the group with BSs (54.4% vs. 28.9%, 29.3% vs. 12.8%, and 11.6% vs. 5.0%, respectively; all *p* < 0.001). Similarly, the intake of antiplatelet drugs, anticoagulants, and iron supplements was more prevalent in this group (8.7% vs. 1.6%, 17.6% vs. 4.4%, and 8.5% vs. 4.0%, respectively; all *p* < 0.001). Comparing the use of vonoprazan and other PPIs, it was found that in cases with BSs, the use of vonoprazan alone, PPIs (excluding vonoprazan), and both drugs were 4.3%, 27.0%, and 23.0% (21/130/111), respectively. In contrast, in cases without BSs, the rates were 4.5%, 14.6%, and 9.8% (282/920/620), respectively, indicating a significant association between the use of PPIs other than vonoprazan and the presence of BSs. In terms of laboratory testing, patients with BSs exhibited higher serum creatinine (0.9 vs. 0.8 mg/dL; *p* < 0.001) and lower hemoglobin levels (12.4 vs. 12.9 g/dL; *p* < 0.001), while no significant differences were observed for serum iron and serum ferritin (*p* = 0.6 and *p* = 0.3 respectively). Additionally, endoscopic findings indicated a higher prevalence of gastric atrophy in patients with BSs at 66.8%, compared with 52.6% in those without BSs, and a lower occurrence of current *H. pylori* infection in the patients with BSs at 1.0% vs. 9.8% in those without BSs, with both observations showing statistical significance (*p* < 0.001). The subgroup analysis of the diffuse group (those with >10 BSs in the stomach) indicated a higher degree of atrophy (open type/closed type/none: 44.3%/32.8%/22.9% vs. 31.0%/29.0%/40.0%; *p* < 0.001) and a higher prevalence of the diffuse pattern post‐*H. Pylori* eradication (51.0% vs. 41.0%; *p* = 0.001; Table S2).

**TABLE 2:** Comparing patients with and without black spots in univariate analysis.

	BSs present, *n* = 482	BSs absent, *n* = 6296	*p*‐value
Sex, *n* (%)			0.148
Male	299 (62.0)	3613 (57.4)	
Female	183 (38.0)	2683 (42.6)	
Age, years (median [IQR])	75.0 (68.0–80.0)	69.0 (55.0–76.0)	<0.001
H2 blocker use, *n* (%)	39 (8.1)	342 (5.4)	0.018
PPI use, *n* (%)	262 (54.4)	1822 (28.9)	<0.001
Vonoprazan, n (%)	21 (4.3)	282 (4.5)	
PPIs (excluding vonoprazan), *n* (%)	130 (27.0)	920 (14.6)	
Both drugs, *n* (%)	111 (23.0)	620 (9.8)	
Statin use, *n* (%)	141 (29.3)	808 (12.8)	<0.001
Corticosteroid use, *n* (%)	56 (11.6)	317 (5.0)	<0.001
Antiplatelet drug use, *n* (%)	42 (8.7)	101 (1.6)	<0.001
Anticoagulant drug use, *n* (%)	85 (17.6)	274 (4.4)	<0.001
Iron preparation use, *n* (%)	41 (8.5)	249 (4.0)	<0.001
Atrophic border, *n* (%)			
None	160 (33.2)	2966 (47.4)	<0.001
Closed‐type	147 (30.5)	1376 (22.0)	
Open‐type	175 (36.3)	1911 (30.6)	
Hp infection status, *n* (%)			<0.001
None	158 (32.8)	2905 (46.1)	
Past infection	217 (45.0)	1238 (19.7)	
Current infection	5 (1.0)	618 (9.8)	
Unknown	102 (21.2)	1535 (24.4)	
Cr, mg/dL (median [IQR])^†^	0.9 (0.7–1.3)	0.8 (0.7–1.0)	<0.001
Hb, g/dL (median [IQR])^†^	12.4 (10.6–13.8)	12.9 (11.2–14.2)	<0.001
Serum iron, µg/dL (median [IQR])^†^	68.0 (40.5–96.5)	70.0 (43.0–100.8)	0.641
Serum ferritin, ng/mL (median [IQR])^†^	57.0 (22.0–193.0)	48.0 (19.0–120.0)	0.328
Chol, mg/dL (median [IQR])^†^	188.0 (160.0–218.3)	191.0 (164.0–216.0)	0.416
HbA1c, % (median [IQR])^†^	6.0 (5.6–6.6)	5.9 (5.6–6.5)	0.259

Abbreviations: Chol, cholesterol; Cr, creatinine; Hb, hemoglobin; HbA1c, hemoglobin A1c; Hp, *Helicobacter pylori*; IQR, interquartile range; PPI, proton pump inhibitor.

^†^ The test values displayed are based on measurements from the following number of patients: Cr from 400/5389 (with/without BSs), Hb from 404/5426, serum iron from 71/1014, serum ferritin from 51/599, chol from 200/2401, and HbA1c from 172/2048 patients. Patients whose data is not shown did not undergo the testing, and the data were not collected.

### Multivariate analysis

**FIGURE 2:**
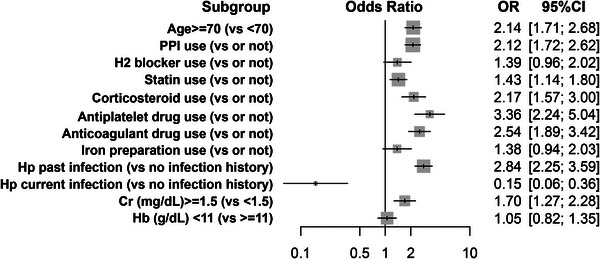
Multivariate analysis comparing the patient outcomes with and without black spots. IQR, interquartile range; PPI, proton pump inhibitor; Hp, *Helicobacter pylori*; Cr, creatinine; Hb, hemoglobin; OR, odds ratio; CI, confidence interval.

In the multivariate analysis, which was built upon the univariate findings, patient data were stratified by clinically significant benchmarks, such as aged ≥70 years, creatinine levels >1.5 mg/dL indicative of chronic renal failure, and hemoglobin levels <11 g/dL suggestive of anemia. The multivariate analysis extracted several risk factors for the presence of BSs in the stomach (Figure 2 and Table S3). Specifically, those aged ≥70 years (odds ratio [OR] 2.13; 95% confidence interval [CI] 1.70–2.67) and use of PPIs (OR 2.15; 95% CI 1.74–2.66), statins (OR 1.72; 95% CI 1.36–2.18), corticosteroids (OR 2.13; 95% CI 1.53–2.96), antiplatelets (OR 3.32; 95% CI 2.21–4.98), and anticoagulants (OR 2.48; 95% CI 1.84–3.35) were all significant for the presence of BSs. Additionally, a previous history of *H. pylori* infection was a notable risk factor for the presence of BSs (OR 2.79; 95% CI 2.21–3.53), whereas an ongoing *H. pylori* infection appeared to reduce the risk of BSs (OR 0.15; 95% CI 0.06–0.36). Elevated creatinine levels (>1.5 mg/dL) were also linked to an increased risk of BSs in the stomach (OR 1.69; 95% CI 1.26–2.27).

### NanoSuit method proved localization of iron

**FIGURE 3:**
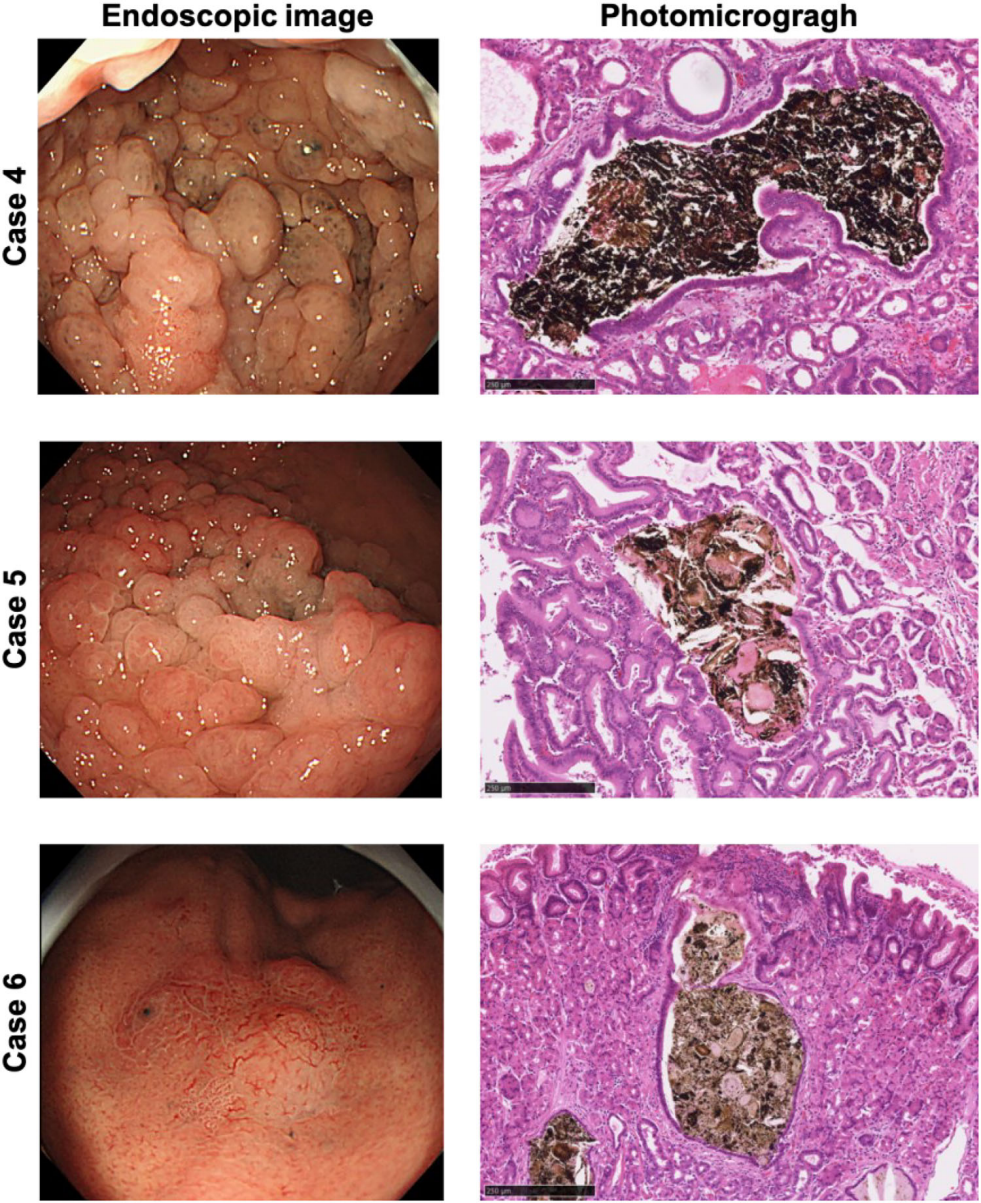
Endoscopic and optical microscopic findings of black spots. Images in the left column are endoscopic images of black spots. Cases 4 and 5 show black spots inside fundic gland polyps, and Case 6 shows black spots inside gastric adenocarcinoma of fundic gland type. The image in the right column is from a light microscope. Brownish deposits are seen inside the fundic gland cysts. Scale bar = 250 µm.

Among the patients with endoscopically observed BSs, biopsies were taken from the BSs in 10, and surgical treatment was performed in 35. Of these, 11 patients exhibited brownish deposits within the dilated fundus gland (Table 3 and Figure 3). Specifically, these instances comprised five cases within fundic gland polyps against a familial adenomatous polyposis background, one within gastric adenocarcinoma of fundic‐gland type, three within other fundic gland polyps, and two within normal mucosa.

**TABLE 3:** Background characteristics of patients undergoing the NanoSuit‐correlative light and electron microscopy method.

Case no.	Sex	Age	Endoscopic deposited area	Background disease	*H. pylori* eradication	PPIs	Statins	Corticosteroids	Antiplatelet drugs	Anticoagulant drugs	Tissue sample
1	Female	58	FGP	FAP	No	Yes	No	No	No	No	Ope
2	Female	56	FGP	FAP	No	No	No	No	No	No	Ope
3	Female	37	FGP	FAP	No	Yes	No	No	No	No	Ope
4	Female	66	FGP	FAP	No	Yes	No	No	No	No	Ope
5	Male	48	FGP	FAP	No	No	No	No	No	No	Ope
6	Male	71	Tumor	GA‐FG	Yes	No	No	No	No	No	Ope
7	Male	72	Normal mucosa	EGJC	Yes	No	Yes	No	No	No	Ope
8	Male	76	FGP	None	No	Yes	Yes	Yes	Yes	No	EMR
9	Male	83	Normal mucosa	GC	Yes	Yes	No	No	Yes	No	Ope
10	Male	82	FGP	None	No	Yes	No	Yes	No	No	biopsy
11	Female	75	FGP	None	No	Yes	No	No	Yes	Yes	biopsy

Abbreviations: EGJC, esophagogastric junction cancer; EMR, endoscopic mucosal resection; FAP, familial adenomatous polyposis; FGP, fundic gland polyp; GA‐FG, gastric adenocarcinoma of fundic gland type; GC, gastric cancer; Ope, surgical operation; PPI, proton pump inhibitor.

**FIGURE 4:**
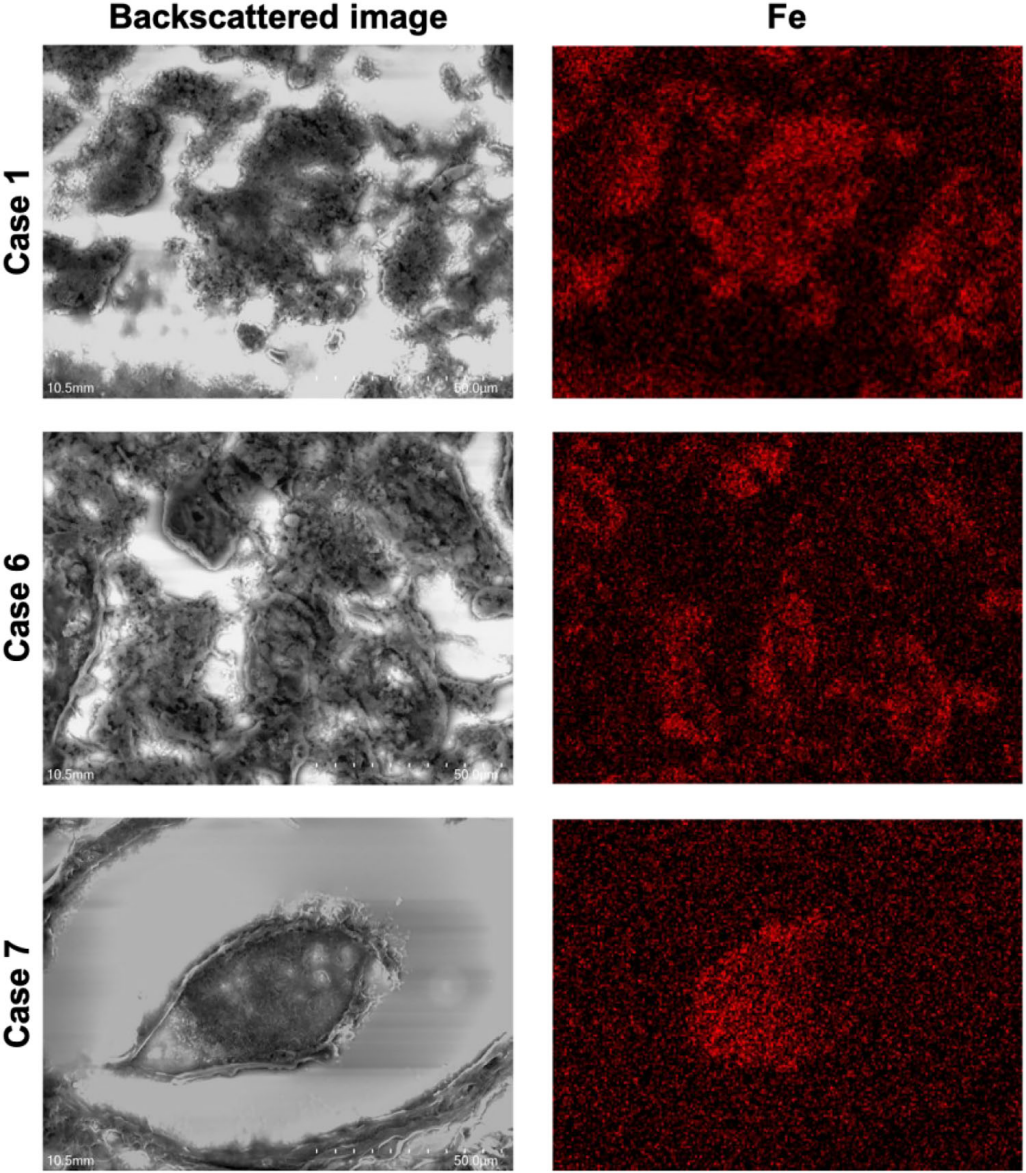
Visualization of deposits in fundic gland cysts using scanning electron microscopy‐energy dispersive X‐ray spectroscopy analysis with the NanoSuit‐correlative light and electron microscopy method. Images in the left column are backscattered scanning electron microscopy images showing bright areas in the deposits. The right column images are elemental mapping images using the NanoSuit‐correlative light and electron microscopy) method combined with scanning electron microscopy‐energy dispersive X‐ray spectroscopy analysis showing iron deposition.

**FIGURE 5:**
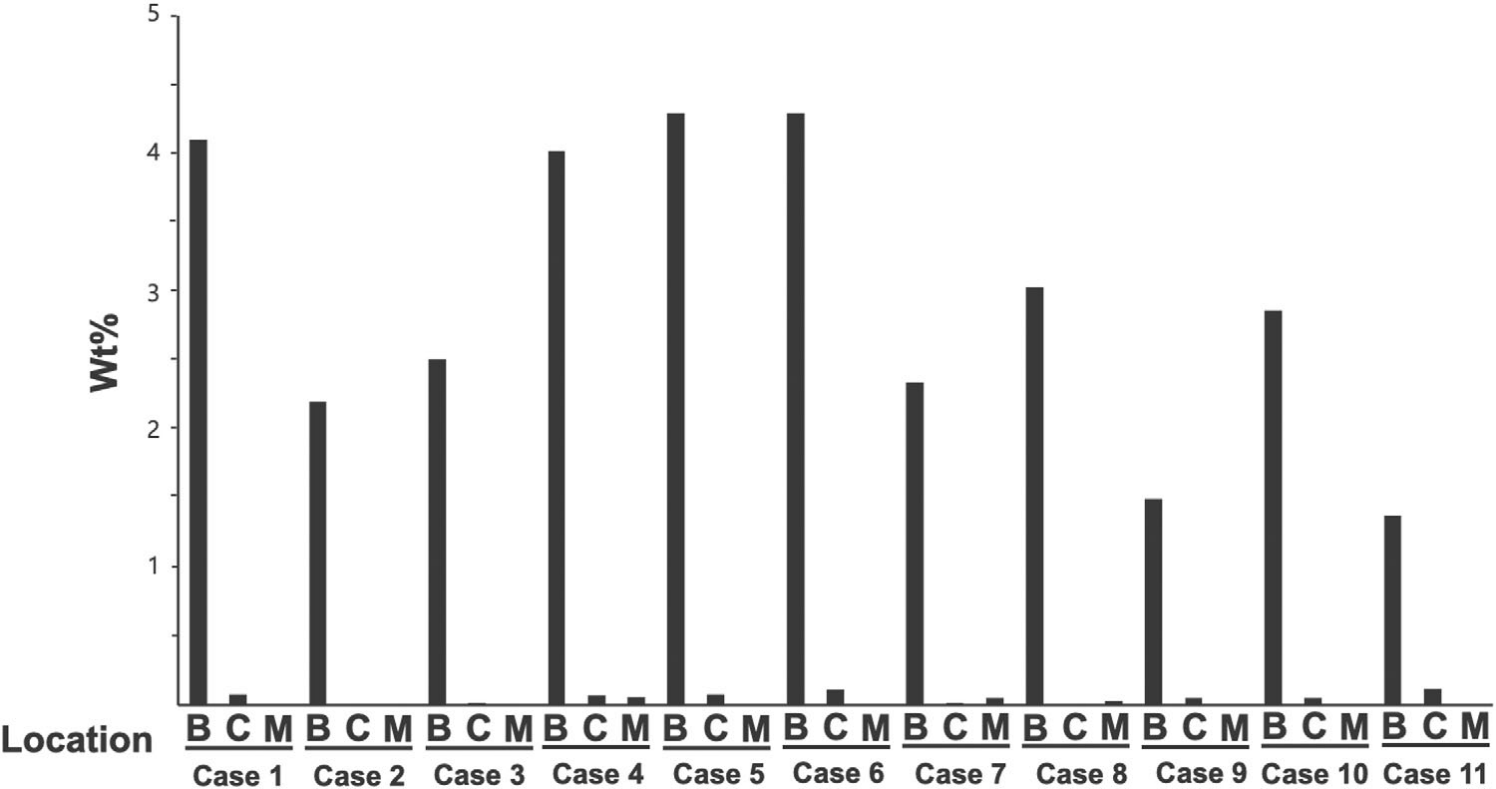
Comparison of iron weight percent in the black spots. Spectral analysis between bright area (B), fundic gland cysts (C), and normal mucosa (M) by SEM‐EDS (scanning electron microscopy‐energy dispersive X‐ray spectroscopy) using the NanoSuit‐CLEM (correlative light and electron microscopy) method. A comparison of the weight percentage (wt%) of iron is obtained by spectrum analysis.

The regions containing the brown deposits were examined using the NanoSuit‐CLEM method. The backscattered SEM images consistently revealed granular, bright contrast areas in all cases (Figure 3 and Figure S1). Notably, elemental mapping via SEM‐EDS pinpointed the presence of iron (Fe) in these areas. Moreover, the weight percent (wt%) of iron within the BS regions was significantly higher compared to the background, and interestingly, iron localization was not observed in dilated gland ducts or normal mucosa without BSs (Figures 4 and 5). The distribution of elements other than iron is detailed in Table S4.

## DISCUSSION

In this research, we conducted a comprehensive analysis of patients with BSs in the stomach and demonstrated that a) BSs are less likely to occur in the presence of active *H.pylori* infection and b) iron is specifically deposited within BSs. To our knowledge, these findings are being reported for the first time, marking a significant contribution to understanding BSs in the stomach.

The clinicopathological analysis revealed that 7.1% of the patients undergoing upper gastrointestinal endoscopy exhibited gastric BSs. These BSs were particularly prevalent among specific patient groups, manifesting in 12.6% (262 out of 2084) of those on PPIs and 14.9% (217 out of 1455) of patients post‐*H. Pylori* eradication. Conversely, BSs were present in only 0.8% (five out of 623) of patients with active *H. pylori* infection, particularly in only 0.5% (one out of 192) of patients with diffuse BSs. The observed pattern in the study suggests that the presence of gastric BSs might indicate inactive or absent *H. pylori*‐related inflammation. This relationship proposes that BSs could potentially serve as a non‐invasive clinical marker for *H. pylori* inflammation, identifiable through endoscopic observation. Furthermore, when comparing vonoprazan with PPIs, it was observed that PPIs were significantly more likely to be associated with BSs. Specifically, 12.3% (130 out of 1050) of patients on PPIs alone experienced BSs compared with 6.9% (21 out of 303) of patients on vonoprazan alone. These findings align with those from the VISION trial and suggest that the differing likelihood of BS occurrence between PPIs and vonoprazan could stem from variations in their mechanisms of acid suppression.^17^

The etiology behind gastric BSs has been elusive, but this study sheds light on potential mechanisms. First, dilation of the gastric fundic glands appears to be a precursor, potentially initiated by hypergastrinemia due to PPI use. This condition is hypothesized to induce morphological alterations in gastric wall cells, leading to obstruction at the narrowed sections, increased outflow resistance, and, consequently, cystic dilation of the gastric fundic glands.^18^, ^19^, ^20^ PPI usage has also been associated with heightened expression of aquaporin‐4 in the wall cells from the basal to the narrow part of the fundus gland, potentially facilitating water migration into the gland lumen, promoting gastric fundus gland dilation, and inducing morphological changes in gastric wall cells.^21^, ^22^ Moreover, corticosteroid administration and renal impairment, known to elevate serum gastrin levels, might contribute to gastric fundic gland dilation.^23^, ^24^ It is also posited that proteases incited by *H. pylori* infection may expedite the degradation and efflux of gastric mucus, impeding cystic dilation.^18^ Consistent with this notion, the current study observed a diminished risk of BSs in association with *H. pylori* infection, aligning with the hypothesis that *H. pylori* eradication restores basal gastric gland function and enhances gastric acid secretion, potentially leading to fundic gland dilation.^25^, ^26^

Secondly, iron deposition in the dilated fundic gland is noted. The presence of iron in gastric juice is a well‐documented phenomenon, and prolonged retention of gastric juice within the dilated fundus gland might amplify the iron concentration, leading to its precipitation.^27^, ^28^ Hemorrhage within the dilated fundus gland could further escalate iron levels. This theory is corroborated by the increased risk of BSs associated with antithrombotic drug use. Additionally, statin usage, implicated in inducing ferroptosis and iron accumulation in adipocytes and cardiomyocytes, may similarly provoke iron accumulation in gastric mucosal cells, contributing to elevated iron concentrations in cysts.^29^, ^30^

In prior research where the NanoSuit‐CLEM method was applied to pathological specimens with siderosis, intracellular iron deposition was confirmed.^9^ This type of iron deposition is also characteristic of diseases like hemochromatosis, which involves extensive iron accumulation primarily in hepatocytes, and aceruloplasminemia, where iron predominantly accumulates in the neuronal cells of the brain.^31^, ^32^ These disorders are marked by iron accumulation within cells. However, conditions leading to iron deposition within luminal spaces, as seen in the case of BSs, are relatively rare. This contrast highlights the uniqueness of the iron deposition pattern observed in BSs, differing significantly from the more common intracellular iron accumulation in other pathologies.

This research provides valuable insights but also has inherent limitations. The findings from this highly specialized, single‐center, retrospective study, conducted at a university hospital, may have limited generalizability. The analysis was confined to stored endoscopic images, potentially leading to an underestimation of the actual prevalence of BSs in areas where images were not archived. The medication histories, while meticulously extracted from medical records, might not entirely capture the patients' full medication profiles. Furthermore, consistent with previous research, endoscopic biopsies from areas with BSs frequently result in unclear pathological findings.^33^ This trend was also observed in the current study. Most cases (nine out of 11) subjected to the NanoSuit‐CLEM method were based on surgical resections. This reliance on surgically resected specimens instead of biopsy samples might influence the study findings. Despite these challenges, the uniform detection of iron in all cases examined with the NanoSuit‐CLEM method is a significant and noteworthy discovery.

## CONCLUSIONS

The study results suggest a negative correlation between the presence of BSs and active *H. pylori* infection. Consistent with previous findings, PPIs and a history of *H. pylori* eradication have been identified as risk factors. Additionally, this study has revealed that antithrombotic drug use also poses a risk. A critical aspect of this research is the use of the NanoSuit‐CLEM method combined with SEM‐EDS, which has successfully detected iron deposition within these BSs. This discovery provides significant insights into the potential causes of these BSs.

## CONFLICT OF INTEREST STATEMENT

None.

## ETHICS STATEMENT

The study was approved by the Institutional Review Board of the Hamamatsu University School of Medicine (approval no. 22‐014, 23–239), which confirmed that the study complied with the ethical guidelines of the Helsinki Declaration.

## PATIENT CONSENT STATEMENT

Written informed consent was obtained from patients for participation in the study. In situations where obtaining written consent was not feasible, an opt‐out procedure was implemented to ensure ethical compliance and respect for patient autonomy.

## Supporting information


**TABLE S1**: Drug class and medication name.
**TABLE S2**: Univariate analysis comparing whether the black spots are diffuse or not.
**TABLE S3**: Multivariate analysis comparing the patient outcomes with and without black spots.
**TABLE S4**: List of weight percentage (wt%) of elements detected by SEM‐EDS analysis using the NanoSuit‐CLEM method.
**FIGURE S1**: Visualization of deposits in fundic gland cysts using SEM‐EDS analysis with the NanoSuit‐CLEM method.
