# Supplementary material for: Iron deposition in gastric black spots: Clinicopathological insights and NanoSuit‐correlative light and electron microscopy analysis
Source: DEN Open. 2024 Jun 17;5(1):e398. doi: 10.1002/deo2.398 (PMC11182783; doi:10.1002/deo2.398)
Supplement: Supplementary file 1 — TABLE S1: Drug class and medication name. TABLE S2: Univariate analysis comparing whether the black spots are diffuse or not. TABLE S3: Multivariate analysis comparing the patient outcomes with and without black spots. TABLE S4: List of weight percentage (wt%) of elements detected by SEM‐EDS analysis using the NanoSuit‐CLEM method. FIGURE S1: Visualization of deposits in fundic gland cysts using SEM‐EDS analysis with the NanoSuit‐CLEM method. [file DEO2-5-e398-s001.docx]

**Table S1.** Drug class and medication name

| Drug class | Medication name |
| --- | --- |
| H2 blockers | Roxatidine |
|  | Famotidine |
|  | Cimetidine |
|  | Lafutidine |
|  | Nizatidine |
| PPIs | Omeprazole |
|  | Lansoprazole |
|  | Rabeprazole |
|  | Esomeprazole |
|  | Vonoprazan |
| Statins | Pravastatin |
|  | Simvastatin |
|  | Rosuvastatin |
|  | Pitavastatin |
|  | Atorvastatin |
| Corticosteroids | Hydrocortisone |
|  | Dexamethasone |
|  | Betamethasone |
|  | Prednisolone |
|  | Methylprednisolone |
| Antiplatelet drugs | Ticlopidine |
|  | Clopidogrel |
|  | Cilostazol |
|  | Eicosapentaenoic Acid Ethyl |
|  | Aspirin |
|  | Prasugrel |
| Anticoagulant drugs | Warfarin |
|  | Dabigatran |
|  | Edoxaban |
|  | Rivaroxaban |
|  | Apixaban |
| Iron preparations | Ferrous Fumarate |
|  | Dried Ferrous Sulfate |
|  | Ferrous Citrate |

PPI, proton pump inhibitor

**Table S2.** Univariate analysis comparing whether the black spots are diffuse or not

|  | Diffuse condition, n=192 | Non-diffuse condition, n=290 | p value |
| --- | --- | --- | --- |
| Sex, n (%) |  |  | 0.632 |
| male | 122 (63.5) | 177 (61.0) |  |
| female | 70 (36.5) | 113 (39.0) |  |
| Age, years (median (IQR)) | 76.0 (68.0-81.0) | 74.0 (68.3-79.0) | 0.326 |
| H2 blocker use, n (%) | 16 (8.3) | 23 (7.9) | 0.866 |
| PPI use, n (%) | 105 (54.7) | 157 (54.1) | 0.926 |
| Statin use, n (%) | 53 (27.6) | 88 (30.3) | 0.541 |
| Corticosteroid use, n (%) | 22 (11.5) | 34 (11.7) | 1 |
| Antiplatelet drugs use, n (%) | 33 (17.2) | 52 (17.9) | 0.903 |
| Anticoagulant drugs use, n (%) | 22 (11.5) | 20 (6.9) | 0.099 |
| Iron preparation use, n (%) | 20 (10.4) | 21 (7.2) | 0.245 |
| Atrophic border, n (%) |  |  |  |
| None | 44 (22.9) | 116 (40.0) | <0.001 |
| Closed-type | 63 (32.8) | 84 (29.0) |  |
| Open-type | 85 (44.3) | 90 (31.0) |  |
| Hp infection status, n (%) |  |  | <0.001 |
| None | 44 (22.9) | 114 (39.3) |  |
| Past infection | 98 (51.0) | 119 (41.0) |  |
| Current infection | 1 (0.5) | 4 (1.4) |  |
| Unknown | 49 (25.5) | 53 (18.3) |  |
| Cr, mg/dL (median [IQR]) | 1.0 (0.8-1.4) | 0.9 (0.7-1.2) | 0.126 |
| Hb, g/dL (median [IQR]) | 12.6 (10.5-13.8) | 12.4 (10.7-13.7) | 0.681 |
| Serum iron, μg/dL (median [IQR]) | 62.0 (40.3-99.0) | 68.0 (43.0-88.0) | 0.867 |
| Serum ferritin, ng/mL (median [IQR]) | 38.0 (15.8-145.3) | 60.0 (23.0-203.0) | 0.554 |
| Chol, mg/dL (median [IQR]) | 187.0 (158.5-220.0) | 189.0 (160.0-218.0) | 0.629 |
| HbA1c, % (median [IQR]) | 6.0 (5.7-6.7) | 5.9 (5.6-6.5) | 0.118 |

IQR, interquartile range; PPI, proton pump inhibitor; Hp, *Helicobacter pylori*; Cr, creatinine; Hb, hemoglobin; Chol, cholesterol; HbA1c, hemoglobin A1c

**Table S3:** Multivariate analysis comparing the patient outcomes with and without black spots

|  | Multivariate analysis | |
| --- | --- | --- |
|  | OR (95% CI) | p value |
| Age (1-year increments) | 1.04 (1.03-1.05) | <0.001 |
| H2 blocker use (vs. no use) | 1.32 (0.89-1.95) | 0.16 |
| PPI use (vs. or no use) | 2.15 (1.71-2.69) | <0.001 |
| Statin use (vs. or no use) | 1.66 (1.30-2.12) | <0.001 |
| Corticosteroid use (vs. or no use) | 2.28 (1.62-3.19) | <0.001 |
| Antiplatelet drug use (vs. or no use) | 3.13 (2.05-4.79) | <0.001 |
| Anticoagulant drug use (vs. or no use) | 2.41 (1.78-3.28) | <0.001 |
| Iron preparation use (vs. or no use) | 1.41 (0.95-2.10) | 0.09 |
| Hp past infection (vs. no infection history) | 2.62 (2.02-3.38) | <0.001 |
| Hp current infection (vs. no infection) | 0.16 (0.06-0.43) | <0.001 |
| Cr (1 mg/dL increments) | 1.12 (1.06-1.19) | <0.001 |
| Hb (1 g/dL increments) | 1.01 (0.96-1.06) | 0.7 |

PPI, proton pump inhibitor; Hp, *Helicobacter pylori*; Cr, creatinine; Hb, hemoglobin; OR, odds ratio; CI, confidence interval

**Figure S1:** Visualization of deposits in fundic gland cysts using SEM-EDS analysis with the NanoSuit-CLEM method

In cases 2–5 and 8-11, deposits within FGCs are shown by SEM-EDS analysis using the NanoSuit-CLEM method. The results, apart from those in Figure 4, are shown in this figure. Images in the left column are backscattered SEM images showing a bright area. The right column images are elemental mapping images using SEM-EDS analysis showing iron deposition (Fe).

**Table S4.** List of weight percentage (wt%) of elements detected by SEM-EDS analysis using the NanoSuit-CLEM method

| **Case** | **Location ^1^** | **Element ^2^** | | | | | | |
| --- | --- | --- | --- | --- | --- | --- | --- | --- |
|  |  | **Fe** | **C** | **Ca** | **Mg** | **Na** | **O** | **Si** |
| No. 1 | B | 4.1 | 65.2 | 1.2 | 0.6 | 2 | 17.9 | 8.7 |
|  | C | <0.1 | 35.4 | 2.7 | 1.7 | 5.6 | 33.1 | 20.9 |
|  | M | <0.1 | 64.9 | 1.8 | 0.6 | 1.8 | 21.4 | 7.9 |
| No. 2 | B | 2.2 | 47.7 | 1.8 | 1.1 | 3.7 | 28.9 | 14.3 |
|  | C | <0.1 | 17.2 | 4.3 | 2.1 | 6.4 | 39.7 | 29.7 |
|  | M | <0.1 | 54.6 | 3.2 | 1.3 | 3.3 | 15.8 | 21.2 |
| No. 3 | B | 2.5 | 61.5 | 2.1 | 0.8 | 2.1 | 17.7 | 12.9 |
|  | C | <0.1 | 17.8 | 4.3 | 2.2 | 6.4 | 39.1 | 29.6 |
|  | M | <0.1 | 60.3 | 1.7 | 0.7 | 2.2 | 24.3 | 9.5 |
| No. 4 | B | 4 | 68.5 | 1.3 | 0.4 | 1.2 | 16.9 | 7.4 |
|  | C | <0.1 | 34.3 | 2.8 | 1.6 | 5.5 | 34.3 | 21.1 |
|  | M | <0.1 | 74.4 | 0.9 | 0.2 | 0.7 | 20.2 | 2.9 |
| No. 5 | B | 4.3 | 67 | 1.8 | 0.7 | 1.8 | 12.8 | 11.2 |
|  | C | <0.1 | 19.4 | 4.1 | 2 | 6.3 | 39.5 | 28 |
|  | M | <0.1 | 66.2 | 1.9 | 0.6 | 1.6 | 18.5 | 9.9 |
| No. 6 | B | 4.3 | 67.6 | 1.1 | 0.3 | 1 | 21.1 | 4 |
|  | C | 0.1 | 69.3 | 1.2 | 0.4 | 1.4 | 20.7 | 5.6 |
|  | M | <0.1 | 63.9 | 2.4 | 0.6 | 1.3 | 20 | 10.6 |
| No. 7 | B | 2.3 | 60.1 | 2.4 | 1 | 2.9 | 14.3 | 16.3 |
|  | C | <0.1 | 43.7 | 3 | 1.5 | 4.6 | 25.9 | 20.8 |
|  | M | <0.1 | 64.3 | 2.1 | 0.6 | 1.8 | 18 | 11.8 |
| No. 8 | B | 3.0 | 59.5 | 1.9 | 0.7 | 2.2 | 21.6 | 9.5 |
|  | C | <0.1 | 13.9 | 4.3 | 2.3 | 7.1 | 42.6 | 29.4 |
|  | M | <0.1 | 56.2 | 1.6 | 0.8 | 2.8 | 27.3 | 10.6 |
| No. 9 | B | 1.5 | 59.0 | 1.6 | 0.8 | 2.8 | 22.8 | 11.1 |
|  | C | 0.1 | 38.2 | 2.8 | 1.4 | 5.1 | 32.2 | 19.6 |
|  | M | <0.1 | 62.3 | 2.4 | 0.7 | 1.7 | 19.1 | 12.5 |
| No. 10 | B | 2.9 | 53.0 | 2.3 | 1.1 | 3.6 | 20.2 | 16.7 |
|  | C | 0.1 | 15.0 | 4.3 | 2.3 | 6.8 | 40.7 | 30.3 |
|  | M | <0.1 | 50.9 | 2.9 | 1.3 | 3.5 | 21.3 | 19.2 |
| No. 11 | B | 1.4 | 53.0 | 1.8 | 1.0 | 3.5 | 25.0 | 13.9 |
|  | C | 0.1 | 18.7 | 4.3 | 2.1 | 6.3 | 38.2 | 29.7 |
|  | M | <0.1 | 56.1 | 3.5 | 0.8 | 2.0 | 18.1 | 17.1 |

^1^ B, bright area; C, fundic gland cyst; M, normal mucosa. ^2^ If wt% > 1.0% is detected in at least one measurement, the element is listed in this table.
